# Neuronal Lipoprotein Lipase Deficiency Alters Neuronal Function and Hepatic Metabolism

**DOI:** 10.3390/metabo10100385

**Published:** 2020-09-28

**Authors:** Kimberley D. Bruce, Evgenia Dobrinskikh, Hong Wang, Ivan Rudenko, Hong Gao, Andrew E. Libby, Sachi Gorkhali, Tian Yu, Andrea Zsombok, Robert H. Eckel

**Affiliations:** 1 Division of Endocrinology, Metabolism, & Diabetes, Denver Anschutz Medical Campus, University of Colorado, Aurora, CO 80045, USA; hong_wang01@yahoo.com (H.W.); ivan.a.rudenko@gmail.com (I.R.); sachigorkhali@gmail.com (S.G.); tian.yu@ucdenver.edu (T.Y.); robert.eckel@cuanschutz.edu (R.H.E.); 2 Department of Medicine, University of Colorado, Denver Anschutz Medical Campus, Aurora, CO 80045, USA; evgenia.dobrinskikh@cuanschutz.edu; 3 Department of Physiology, School of Medicine, Tulane University, New Orleans, LA 70112, USA; hgao1@tulane.edu (H.G.); azsombo@tulane.edu (A.Z.); 4 Department of Biochemistry and Molecular & Cellular Biology, Georgetown University Medical Center, Washington, DC 20057, USA; andrew.libby@georgetown.edu

**Keywords:** lipoprotein lipase, neuronal metabolism, fatty liver, brain-liver axis, FLIM

## Abstract

The autonomic regulation of hepatic metabolism offers a novel target for the treatment of non-alcoholic fatty liver disease (NAFLD). However, the molecular characteristics of neurons that regulate the brain-liver axis remain unclear. Since mice lacking neuronal lipoprotein lipase (LPL) develop perturbations in neuronal lipid-sensing and systemic energy balance, we reasoned that LPL might be a component of pre-autonomic neurons involved in the regulation of hepatic metabolism. Here, we show that, despite obesity, mice with reduced neuronal LPL (NEXCreLPL^flox^ (LPL KD)) show improved glucose tolerance and reduced hepatic lipid accumulation with aging compared to wilt type (WT) controls (LPL^flox^). To determine the effect of LPL deficiency on neuronal physiology, liver-related neurons were identified in the paraventricular nucleus (PVN) of the hypothalamus using the transsynaptic retrograde tracer PRV-152. Patch-clamp studies revealed reduced inhibitory post-synaptic currents in liver-related neurons of LPL KD mice. Fluorescence lifetime imaging microscopy (FLIM) was used to visualize metabolic changes in LPL-depleted neurons. Quantification of free vs. bound nicotinamide adenine dinucleotide (NADH) and flavin adenine dinucleotide (FAD) revealed increased glucose utilization and TCA cycle flux in LPL-depleted neurons compared to controls. Global metabolomics from hypothalamic cell lines either deficient in or over-expressing LPL recapitulated these findings. Our data suggest that LPL is a novel feature of liver-related preautonomic neurons in the PVN. Moreover, LPL loss is sufficient to cause changes in neuronal substrate utilization and function, which may precede changes in hepatic metabolism.

## 1. Introduction

The liver is central to the pathogenesis of metabolic disorders such as obesity, type 2 diabetes mellitus (T2DM), and cardiovascular disease. It is thought that hepatic lipid accumulation precedes insulin resistance [1,2], which plays a pivotal role in the development of T2DM [3]. Importantly, hepatic neutral lipid accumulation and the incidence of non-alcoholic fatty liver disease (NAFLD) increase markedly with age [4]. Thus, preventing hepatic fat accumulation is a promising strategy to prevent age-associated metabolic disease. The autonomic nervous system (ANS) plays a key role in regulating hepatic metabolism [5] and is, therefore, an attractive target for the treatment of metabolic disease. Metabolic signals from the hypothalamus reach the liver via neuronal pathways that include the brain stem, the sympathetic nerves, and the vagus nerve. However, the metabolic characteristics of hypothalamic preautonomic neurons remain undefined.

The accumulation of intermediary metabolites in specific brain regions can alter hepatic metabolism, suggesting that the brain-liver axis is involved in responding to nutrient status and maintaining whole-body energy homeostasis. For example, central administration of glucose stimulates the conversion of lactate to pyruvate and activates a K_ATP_ channel-dependent pathway within the hypothalamus, leading to lowered very-low density lipoprotein triglycerides (VLDL-TG) secretion from the liver and reduced circulating triglycerides (TG) [6]. In addition, fatty acids (FA) such as oleic acid activate a protein kinase C (PKC-)δ-K_ATP_ channel signaling pathway, resulting in suppressed VLDL-TG secretion in rats [7]. This signaling requires the dorsal vagal complex, highlighting a brain–liver neurocircuitry in which hypothalamic FA sensing triggers a hepatic-neuronal relay to regulate hepatic lipid homeostasis [7]. However, the precise cellular components involved in neuronal FA sensing remain unclear. While several FA transporters have been identified in the brain, these are only associated with specific FA species and do not account for the unique composition of lipid substrates in the brain, i.e., glial-derived lipoproteins. For example, CD36 knock-out mice show reduced monounsaturated FA incorporation into brain phospholipids but no changes in essential polyunsaturated fatty acids (PUFA) concentration [8], suggesting that alternative pathways may regulate PUFA uptake and metabolism.

We and others have previously shown that lipoprotein lipase (LPL)—the rate-limiting enzyme in the hydrolysis of TG-rich lipoproteins and FA uptake—is involved in neuronal lipid uptake and the coordination of systemic metabolism [9,10,11,12,13]. Specifically, mice with neuronal LPL deficiency develop obesity by six months of age and a specific PUFA deficiency associated with neurobehavioral abnormalities [11,13]. Thus, based on the systemic impact of neuronal LPL deficiency, we reasoned that neuronal LPL may play a role in hypothalamic lipid-sensing and the ANS regulation of hepatic metabolism. In the present study, we demonstrate that obese mice with neuronal LPL deficiency show improved glucose tolerance with aging, involving reduced hepatic lipid accumulation and altered activity of liver-related neurons in the PVN. Moreover, LPL depletion is sufficient to alter neuronal substrate metabolism in vitro and in vivo, suggesting that LPL-dependent changes to neuronal metabolic flux may precede changes in systemic metabolism.

## 2. Results

### 2.1. Mice with Neuron-Specific LPL Depletion Have Improved Glucose Tolerance

**Figure 1:**
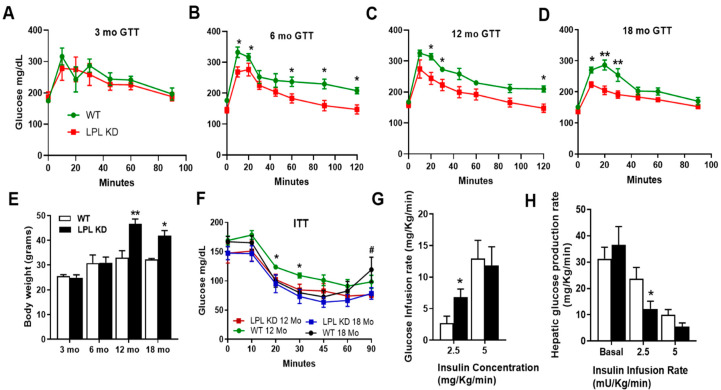
Neuronal lipoprotein lipase (LPL) deficiency improves glucose tolerance with aging. (**A**–**D**) Measurements of glucose tolerance in (NEXCreLPL^flox^) LPL KD versus WT (LPL^flox^) mice at 3 (**A**), 6 (**B**), 12 (**C**), and 18 (**D**) months. (**E**) The body weights of LPL KD (black) and WT (white) mice before terminal experiments at each age point. (**F**) Intraperitoneal (IP) insulin tolerance tests (ITT) in 12 and 18 months in WT mice. (**G**) Steady state whole body glucose infusion rate in LPL KD (black) mice compared to WT (white). (**H**) Endogenous glucose production (EGP) rate in LPL KD mice compared to WT controls. (Where **#** = *p* < 0.1, * = *p* < 0.05, and ** = *p* <0.01 vs. aged-matched WT controls).

To determine whether neuronal LPL deficiency could influence peripheral glucose homeostasis, we performed 2 h intraperitoneal (IP) glucose tolerance tests (GTT) in LPL KD (NEXCreLPL^flox^) and WT (LPL^flox^) mice at 3, 6, 12, and 18 months of age (Figure 1A–D). LPL KD mice showed improved glucose clearance by 6 months, with lower plasma glucose concentrations throughout the study (*p* < 0.05 versus WT) (Figure 1B). LPL KD mice showed lower plasma glucose concentrations at 12 and 18 months compared to WT mice (Figure 1C,D). Importantly, LPL KD mice were markedly heavier than controls by 12 months (Figure 1E).

### 2.2. LPL KD Mice Have Reduced Hepatic Glucose Production

Since neuronal LPL deficiency is associated with improved glucose tolerance despite obesity, we reasoned that these animals may be more insulin sensitive. To test this, mice were given an IP bolus of insulin at 12 and 18 months. Although both WT and LPL KD mice responded to the insulin bolus, this response was greater in the 12 months LPL KD mice compared to WT (*p* < 0.05 at 20 and 30 min) and longer lasting in the 18 months LPL KD mice, with a trend for plasma glucose levels rising more rapidly in the WT controls (*p* < 0.09) (Figure 1F).

In patients with T2DM, endogenous glucose production (EGP) is the primary factor responsible for elevations in fasting plasma glucose levels and contributes importantly to postprandial hyperglycemia. Thus, we hypothesized that the age-associated improvements in glucose tolerance observed in the LPL KD mice may be due to lower EGP. Hence, we performed hyperinsulinemic-euglycemic clamps to show that hepatic but not systemic insulin sensitivity was increased in LPL KD mice compared to WT mice, an effect demonstrated at low physiological insulin infusion rates [14], i.e., 2.5 mg/kg/min (Figure 1G). In addition, LPL KD mice showed an increased ability of insulin to suppress EGP at low insulin concentrations (Figure 1H).

### 2.3. Neuronal LPL-KD Mice Have Reduced Hepatic Lipid Accumulation

**Figure 2:**
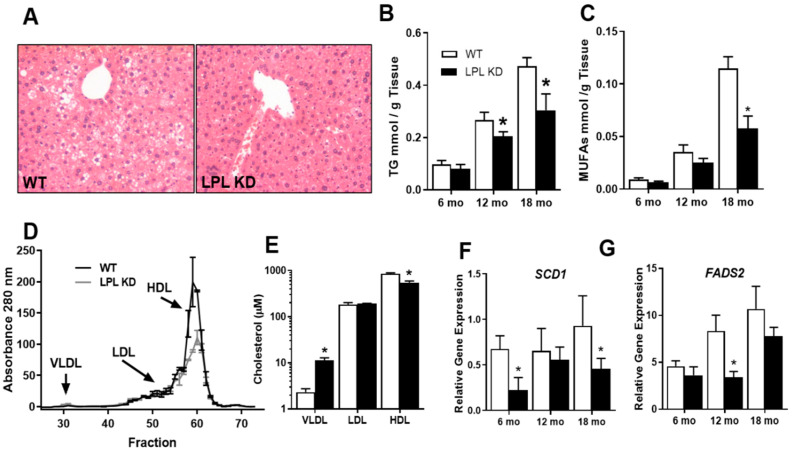
Hepatic lipid accumulation is reduced in neuron-specific LPL KD mice. (**A**) Hematoxylin and eosin stained liver of 12 months WT (LPL^flox^) and LPL KD (NEXCreLPL^flox^) mice. (**B**) Hepatic triglyceride (TG) concentration in LPL KD (black bars) compared to WT mice (white bars) at 6, 12, and 18 months. (**C**) Hepatic mono-unsaturated fatty acid (MUFA) concentration reduced in LPL KD mice compared to WT mice at 6, 12, and 18 months. (**D**) Fast protein liquid chromatography (FPLC) fractionation of lipoproteins from plasma of LPL KD and WT mice at 18 months. (**E**) Cholesterol content of lipoprotein fractions at 18 months. (**F**,**G**) Relative gene expression of *SCD1* and *FADS2* at 6, 12, and 18 months. (* = *p* < 0.05 vs. aged-matched WT controls).

Since hepatic lipid accumulation can precede changes in hepatic insulin sensitivity [15], we hypothesized that changes in glucose tolerance and insulin sensitivity in the LPL KD mice were a consequence of altered hepatic lipid metabolism. Thus, we performed histological analysis to determine hepatic steatosis in the livers of 12 months WT and LPL KD mice. Lipid droplets were clearly visible in liver sections from WT mice and were present as moderate micro-vesicular steatosis (Figure 2A). However, fewer lipid droplets were observed in liver sections from the LPL KD mice (Figure 2A). We also observed a reduced total TG content in the livers of LPL KD mice at 12 and 18 months (Figure 2B). In fact, this decrease was observed in almost all lipid species (data not shown), with the greatest difference being observed in the monounsaturated (MUFA) content at 18 months (Figure 2C).

Since VLDL export from the liver is a major contributor towards hepatic lipid homeostasis [16], we measured the plasma lipoprotein profile of LPL KD and WT mice at 18 months (Figure 2D). Importantly, as samples were separated by size exclusion, chromatography absorbance was measured at 280 nm. Therefore, the trace shown indicated the protein concentration of the eluted sample and allowed identification of the lipoprotein fractions (Figure 2D). Cholesterol was then subsequently measured for each lipoprotein class. We observed an increase in plasma VLDL-cholesterol concentrations in LPL KD vs. WT mice (*p* < 0.05 vs. WT) (Figure 2E). Of note, low density lipoprotein (LDL) cholesterol concentrations were almost identical between the LPL KD and the WT mice. However, plasma high-density lipoprotein (HDL) cholesterol was lower in the LPL KD mice (*p* < 0.05 vs. WT) (Figure 2E).

To further understand the mechanism behind altered lipid metabolism in the livers of mice lacking neuronal LPL, we measured the expression of genes key to hepatic lipid homeostasis. Stearoyl-CoA desaturase (*SCD1*), the rate-limiting enzyme in MUFA synthesis, was reduced in the LPL KD mice at 6 and 18 months (Figure 2F). In addition, fatty acid desaturase 2 (*FADS2*), which catalyzes the first and rate-limiting step in several fatty acid desaturation pathways, showed a robust increase with age (Figure 2G), which was blunted in the LPL KD mice at 12 months (*p* < 0.05 vs. WT) (Figure 2G).

### 2.4. Liver-Related PVN Neurons of LPL KD Mice Have Reduced Inhibitory Synaptic Control

**Figure 3:**
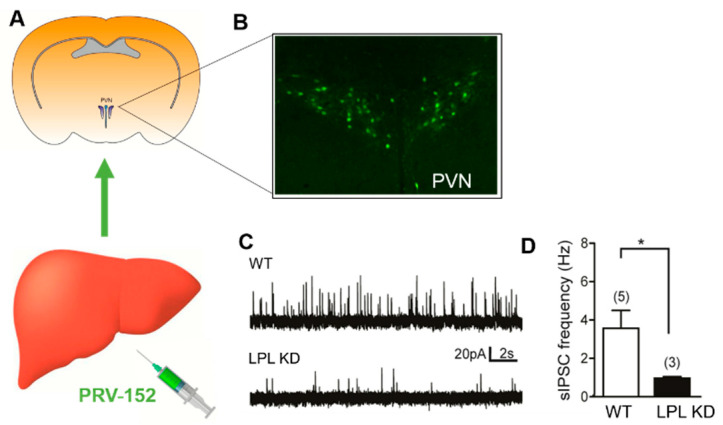
Decreased inhibitory synaptic regulation of liver related paraventricular nucleus (PVN) neurons in LPL KD mice. (**A**,**B**) Liver-related neurons in the PVN were identified with a retrograde transsynaptic viral tracer (PRV-152) and used for patch-clamp recordings. (**C**) Recordings of spontaneous inhibitory post-synaptic currents (sIPSCs) in WT (LPL^flox^) and LPL KD (NEXCreLPL^flox^) mice. (**D**) The frequency of sIPSCs was reduced in LPL KD mice compared to WT mice. (* = *p* < 0.05 vs. WT).

A growing number of studies suggest that the activity of preautonomic neurons in the PVN of the hypothalamus has a pivotal role in the autonomic regulation of hepatic glucose and lipid metabolism [17,18,19,20]. Since synaptic inputs largely contribute to the excitability of neurons, we reasoned that the synaptic control of liver-related neurons in the PVN is altered in mice with neuronal LPL deficiency. Liver-related PVN neurons were identified with the retrograde transsynaptic viral tracer PRV-152 (Figure 3A), and inhibitory and excitatory post-synaptic currents were recorded from enhanced green fluorescent protein (EGFP) expressing PVN neurons. The observed EGFP labeling indicated liver-related preautonomic neurons in the PVN (Figure 3B), consistent with previous findings [17]. Our data showed decreased inhibitory post-synaptic currents (IPSC) frequency in LPL KD mice compared with WT mice (Figure 3C). The average frequency of spontaneous inhibitory post-synaptic currents (sIPSCs) was 0.97 ± 0.07 Hz in LPL KD mice and 3.56 ± 0.93 Hz in WT mice, suggesting that inhibitory neurotransmission to liver-related PVN neurons is reduced in LPL KD mice (*p* < 0.05) (Figure 3D). In contrast, we did not find significant differences in excitatory neurotransmission (*p* > 0.05).

### 2.5. Loss of LPL in PVN Neurons Alters Metabolic Flux

**Figure 4:**
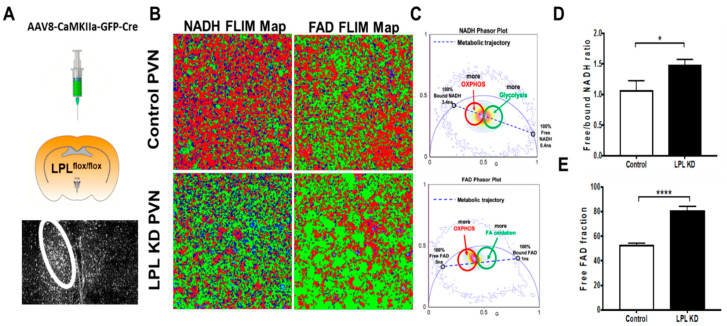
LPL depletion in neurons of the paraventricular nucleus of the hypothalamus (PVN) results in altered neuronal metabolism. (**A**) LPL was depleted in the PVN of LPL^flox^ mice by the delivery of AAV8-CaMKIIa-GFP-Cre, whereas AAV8-CaMKIIa-GFP was administered to control mice. (**B**) Representative images of nicotinamide adenine dinucleotide (NADH) and flavin adenine dinucleotide (FAD) fluorescence lifetime imaging microscopy (FLIM) maps from images of the PVN in control versus LPL KD Mice. (**C**) Phasor plots showing the shift in metabolic trajectory with changing lifetime for NADH and FAD. (**D**) Increased ratio of free/bound NADH in LPL KD PVN neurons. (**E**) Increased free FAD fraction in LPL KD PVN neurons. (* = *p* < 0.05 vs WT, **** = *p* < 0.0001 vs. WT).

We previously showed that neuronal LPL plays an important role in neuronal lipid sensing. However, the mechanism underlying this process remains unknown. Since the canonical role of LPL is to facilitate cellular lipid uptake, we hypothesized that LPL loss might alter neuronal metabolism. A major limiting factor regarding metabolic studies in neurons is the ability to preserve endogenous metabolism at the time of quantification without causing major changes during ex vivo culture. Indeed, many primary cultures of neurons are from the early post-natal period, and endogenous metabolism may differ vastly from that in the adult mouse. Here, we employed fluorescence lifetime imaging microscopy (FLIM) as a novel method to measure the endogenous metabolic status of neurons from fresh-frozen brain tissue with high spatial resolution. LPL was depleted in neurons of the PVN in LPL^flox/flox^ mice using adenoviral delivery of a GFP-Cre virus, driven by the neuronal-specific CamKIIα promoter (Figure 4A). GFP allowed for localization to the PVN (Figure 4A) and to neurons (Figure 4B), which is essential since it is not yet studied how fixation and/or staining alters the ability to measure and discriminate between the lifetimes of NADH and FAD co-enzymes. Overall, we showed that there was a significant shift towards free versus bound NADH in LPL deficient PVN neurons (*p* < 0.05) (Figure 4D), which indicates increased flux through metabolic pathways producing free NADH (e.g., glycolysis), rather than flux through metabolic pathways in which NADH would be bound to metabolic enzymes (e.g., oxidative phosphorylation). Moreover, there was a dramatic shift in the proportion of free FAD versus bound FAD (*p* < 0.001) (Figure 4E), which suggests an increase in processes that produce free FAD (e.g., TCA) and a reduction in processes in which enzymes utilize FAD as a cofactor (e.g., oxidative phosphorylation and FA oxidation).

**Figure 5:**
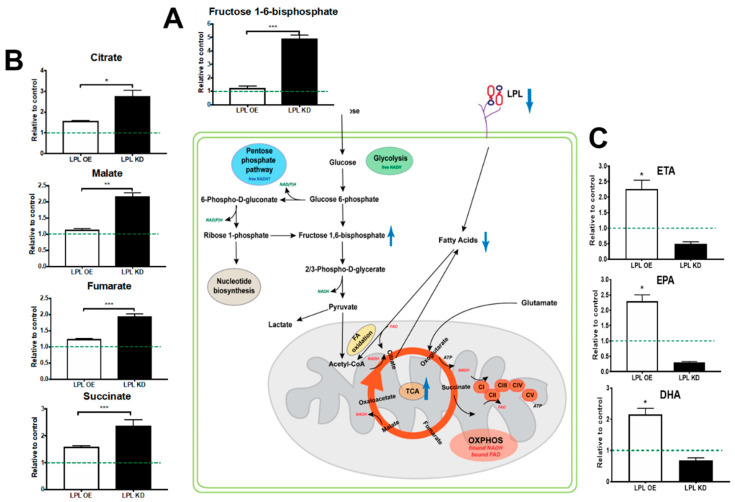
Altered substrate utilization in hypothalamic neurons deficient in or over-expressing LPL. (**A**) LPL KD N41 neurons show increased abundance of fructose 1,6-bisphosphate. (**B**) LPL KD N41 neurons show increased TCA intermediates. (**C**) LPL OE N41 neurons had increased abundance of LC-PUFAs. * = *p* < 0.05 vs. WT, ** = *p* < 0.01 vs. WT, *** = *p* < 0.001 vs. WT.

To validate changes in substrate utilization following LPL loss, we measured global metabolites in hypothalamic neuronal cell lines in which LPL was either depleted (LPL KO N41) or over-expressed (LPL OE N41). Although immortalized or primary cell lines cannot perfectly recapitulate the in vivo studies described above, this established cell tool enables us to validate major changes in metabolic pathways following LPL loss. Here, we found a marked increase in the abundance of fructose 1,6-bisphosphate in LPL KO N41 neurons compared to control neurons (*p* < 0.001) (Figure 5A and Supplementary Figure 1. Although this suggests an increase in glucose utilization following LPL loss via glycolysis, it also indicates increased carbohydrate metabolism, the pentose phosphate pathway (PPP) being the preferred route of glucose utilization in neurons [21]. Importantly, increased glucose utilization increases the production of free NADH, supporting the observation of increased free NADH following LPL loss (Figure 4D). In addition, we found an increase in the abundance of TCA metabolites, such as citrate (*p* < 0.05), malate (*p* < 0.01), fumarate (*p* < 0.001), and succinate (*p* < 0.001) (Figure 5B and Figure S2), in LPL KO N41 neurons, suggesting an augmented flux through this pathway and, thus, increased free FAD production. Additional pathway analysis supported the overrepresentation of TCA metabolites in the LPL KD cells (Figure S4). Interestingly, LC-PUFAs were reduced in the LPL KO N41 neurons but increased in the LPL OE N41 neurons (Figure 5C and Figure S3). While this suggests that FA oxidation flux may be reduced, contributing to free FAD accumulation, it also highlights LPL’s selective role in the uptake of long-chain unsaturated fatty acids, which is consistent with previous studies [10,11,13].

## 3. Discussion

Since aberrant hepatic glucose production and lipid metabolism play a key role in the development of metabolic diseases such as IGT, T2DM, and even hypertriglyceridemia, identification of the mechanisms underlying the brain-liver axis holds promise for the development of novel interventions and therapeutics. Several studies have highlighted the ability of specific neuropeptides and metabolites to act upon key hypothalamic energy-sensing nuclei and, in turn, modulate downstream metabolism [7,22,23,24]. However, how neurons receive such inputs is mostly unknown. The goal of the present study was to investigate whether LPL was involved in the regulation of hepatic metabolism by modulating neuronal metabolism. We reasoned that, since mice with a neuron-specific LPL depletion show hyperphagia, inactivity, and obesity, autonomic regulation of the peripheral metabolic organs such as the liver was likely. Interestingly, we found that, despite obesity, mice with reduced neuronal LPL showed improved glucose tolerance with aging, a phenotype that involves reduced hepatic EGP and preserved hepatic insulin sensitivity despite aging and obesity.

Using GTTs in LPL KD and WT mice at several age points, we found that mice lacking neuronal LPL had improved glucose tolerance compared to WT controls, a key difference that became more pronounced with aging (Figure 1). Importantly, by 12 months, the LPL KD mice were heavier than the WT mice. However, glucose tolerance was preserved throughout aging despite obesity (Figure 1). Although paradoxical, this phenotype is reminiscent of obese individuals who remain “metabolically healthy” [25]. These insulin-sensitive obese (ISO) individuals have lower visceral fat accumulation, less ectopic fat, and less systemic inflammation than insulin-resistant obese individuals [26,27]. Interestingly, LPL KD mice exhibit brown adipose tissue hyperplasia [11] and reduced ectopic hepatic lipid accumulation (Figure 2A), both hallmarks of ISO. In further support, we show that LPL KD mice had improved hepatic insulin sensitivity (Figure. 2). Therefore, it is plausible that a reduction in neuronal LPL influences the autonomic regulation of systemic metabolism to mimic the ISO individuals.

The autonomic regulation of hepatic glucose metabolism is well established. Several studies have demonstrated that stimulation of the splanchnic nerve (sympathetic nervous system (SNS)) modulates liver function, increases EGP, and decreases glycogenesis, whereas stimulating the vagus nerve (parasympathetic nervous system (PNS)) decreases EGP and increases glycogenesis [28,29]. Since we observed lower EGP in mice with reduced neuronal LPL, it is tempting to speculate that we observe increased PNS activity in these animals compared to wild-type mice. However, we cannot rule out the possibility that changes in EGP are due to reduced SNS activity. Thus, further studies are needed to delineate this mechanism. Nonetheless, our findings support the notion that LPL is a feature of preautonomic neurons involved in lipid sensing and the regulation of hepatic glucose metabolism.

The ANS is also involved in the regulation of hepatic lipid metabolism. Here, we show that the abundance of hepatic TG and MUFA (Figure 2C) as well as the expression of *SCD1* (Figure 2F) and *FADS2* (Figure 2G) increase robustly with age in WT mice. This increase in hepatic lipid species is reminiscent of the increase in the prevalence of NAFLD in the aging population [4]. Since the SNS is dysregulated with age [30] and has been implicated in the development of NAFLD [31], our data point to an age-associated increase in SNS activity in WT mice. Importantly, mice with neuronal LPL deficiency have reduced hepatic lipid accumulation and lipogenic gene expression (Figure 2) despite aging. Therefore, it is tempting to speculate that the loss of neuronal LPL dampens an age-associated rise in SNS activity with age. Since we observed a marked decrease in hepatic MUFA and *SCD1*, the enzyme responsible for the formation of mono-unsaturated acyl-CoAs from saturated acyl-CoAs, this highlights a lipogenic pathway regulated by neuronal LPL loss. This is consistent with previous studies in which liver lipids were altered following differential metabolic sensing in the brain. Specifically, central administration of glucose has been shown to mimic the “fed state” in the brain and lower hepatic *SCD1* activity, leading to reduced MUFA synthesis [6].

Preautonomic neurons in the PVN are known to contribute to the ANS regulation of hepatic metabolism [32]. Here, we identified liver-related PVN neurons and determined their synaptic regulation in mice lacking neuronal LPL. Our data clearly showed reduced sIPSCs in liver-related PVN neurons in LPL KD mice (*p* < 0.05 vs. WT) (Figure 3C), suggesting that the inhibitory synaptic regulation of liver-related PVN neurons in these mice is altered. Although further studies are needed to elucidate the mode of neurotransmission, studies have highlighted the role of GABAergic inhibition in the regulation of preautonomic neurons. For example, blockade of GABA_A_ receptors in the PVN caused a pronounced increase in plasma glucose concentration via sympathetic nerves to the liver [20]. This suggests that reduced inhibition of sympathetic PVN neurons may be detrimental to glucose homeostasis. However, at this stage, we could not delineate whether neuronal LPL deficiency resulted in decreased inhibition of pre-sympathetic or pre-parasympathetic neurons.

It is also plausible to suggest that the altered neuronal metabolism could result in changes to synaptic function. To monitor changes in neuronal metabolism ex vivo, we employed FLIM to measure both NADH and FAD lifetime changes following LPL loss. Although it is an established method for measuring endogenous metabolism [33,34], this methodology has not previously been used to further our understanding of neuronal metabolism. Here, we clearly show that free NADH increased in LPL deficient PVN neurons (Figure 4D). Typically, this would suggest an increased flux through glycolysis [35,36,37]. However, since neurons have a limited capacity to upregulate glycolysis [38], increased glucose utilization through the PPP in neurons is more likely [21]. In further support of this interpretation, we also observed a marked increase in fructose 1,6-bisphosphate in LPL-deficient hypothalamic neurons (Figure 5A). Due to the lack of 6-phosphofructo-2-kinase/fructose-2, 6-bisphosphatase-3 (Pfkfb3), fructose-6P could not be shuttled through traditional glycolytic routes and instead was converted to fructose 1,6-bisphosphate and shunted towards the PPP [39]. Therefore, an accumulation of fructose 1,6-bisphosphate suggests an increase in glucose uptake; whether this also suggests a block in the pathway preceding full oxidation of glucose or recycling via gluconeogenesis (unlikely in mammalian neurons) remains unclear. Interestingly, metabolite enrichment analysis suggests that metabolites associated with the TCA and lactose metabolism are up-regulated in the LPL KD cells (Figures S5 and S6). Since lactose synthesis predominantly occurs in the mammary gland, these findings highlight a caveat in non-cell type or tissue specific MetaboAnalyst analysis and are likely due to the enrichment in fructose 1,6-bisphosphate, which is a key metabolite in several pathways of carbohydrate metabolism. The importance of glucose transport in neurons has been demonstrated in several recent studies [40] that challenge the well-established astrocyte-neuron lactate shuttle (ANSL) hypothesis [41] to suggest that glucose is the major substrate for the typical activated brain. Our data suggest that carbohydrate metabolism is increased in LPL depleted neurons. Whether this altered substrate utilization is a compensatory response to reduced lipid uptake following LPL loss is likely but remains to be determined.

FLIM analysis of endogenous neuronal metabolism in PVN neurons also revealed a marked increase in free FAD (Figure 4E). This suggests an increase in pathways in which free FAD would be produced (e.g., complex II activity in the TCA [42]) and reduced “flux” through pathways in which FAD would be enzyme-bound (e.g., FA oxidation). This is supported by the increased TCA cycle metabolites (citrate, malate, fumarate, and succinate) observed in the LPL KD N41 hypothalamic neurons. This suggested partitioning is also consistent with increased glucose uptake and utilization, driving the oxidative metabolism of glucose and/or its intermediary metabolites (i.e., lactate) during reduced FA uptake and oxidation following LPL loss. Importantly, these data represent a single time-point of analysis, and further studies including isotope tracer experiments are warranted to further understand LPL-dependent changes in metabolic flux and neuronal substrate utilization.

Hypothalamic neurons in specific sub-nuclei sense the abundance of leptin, insulin, neuropeptides, and FAs [22,43,44,45], which may result in ATP-dependent potassium channel activation. Although neurons are thought to rely primarily on the oxidative metabolism of glucose/lactate to fulfill bioenergetics needs, fatty acid oxidation may also be important for the metabolism and the function of hypothalamic neurons. For example, the inhibition of Carnitine palmitoyltransferase I (CPT-1) activity in the hypothalamus results in reduced neuronal FA oxidation and increased accumulation of long-chain fatty acids (LC-FAs) [46]. This shift in metabolism may signal a “fed state”, resulting in marked inhibition in food intake and reduced nutrient mobilization, i.e., EGP [46]. It is thought that the accumulation of malonyl-CoA may play a key role in neuronal FA sensing, since inhibition of fatty acid synthase can suppress food intake in a malonyl-CoA dependent manner [47]. In addition, malonyl-CoA is a potent inhibitor of CPT-1 and, thus, LC-FA oxidation. Here, we show that LPL-depleted neurons in the PVN showed increased free NADH and FAD, which is consistent with a metabolic shift away from mitochondrial FA oxidation of LC-FAs (Figure 4D,E). In addition, we demonstrate that neuronal cell lines lacking LPL had diminished accumulation of the LC-PUFAs, whereas neuronal cell lines that overexpressed LPL had increased accumulation of LC-PUFAs, such as eicosatetraenoic acid (ETA), eicosapentaenoic acid (EPA), and docosahexaenoic acid (DHA) (Figure 5C). Importantly, these data recapitulate our findings in vivo, showing reduced LC-PUFA abundance in the brains of mice lacking neuronal LPL [11,13], and highlight the utility of this cell line for investigating underlying metabolic changes following LPL modulation. Moreover, these data suggest that LPL is involved in neuronal uptake of LC-PUFAs, which may be an important signal in the regulation of hepatic metabolism and nutrient mobilization. Findings from existing literature would suggest that neuronal LC-PUFAs depletion may signal poor nutrient availability to stimulate increased food intake and increased EGP [46]. While our data are in part consistent with this notion, these studies have all been acute responses to modulated FA sensing and do not take into account the potential for compensatory mechanisms that occur with aging. For example, it is plausible that long-term changes in essential FA uptake have a larger impact over time than the response to acute changes in nutrient availability. It is well established that LC-PUFAs are found in either the C1 or the C2 position of major phospholipid species that play critical roles in neuronal functions such as growth, signaling, and excitatory and inhibitory synaptic transmission [48]. Therefore, long-term reductions in LPL-mediated LC-PUFA uptake may have profound effects on synaptic transmission and neuronal maintenance over time. Whether these translate to downstream changes in systemic metabolism will be an important focus of future studies.

## 4. Materials and Methods

### 4.1. Animals

All animal procedures were performed in accordance with institutional regulations and the institutional animal care and use ethics Committee at the University of Colorado Anschutz Medical Campus (Protocol #00114, approval date (6 January 2016). Male NEXCreLPL^flox^ (LPL KD) and WT mice were generated as previously described and housed in standard conditions (11). Since NEXCre(+/−) LPL^flox^ and NEXCre(−/−) LPL^flox^ produced similar phenotypes, these mice were grouped (LPL KD) for analysis. Mice were fed standard laboratory chow diet until terminal experiments at 3 months (WT *n* = 4, LPL KD *n* = 5), 6 months (WT *n* = 4, LPL KD *n* = 8), 12 months (WT *n* = 5, LPL KD *n* = 6), and 18 months (WT *n* = 4, LPL KD *n* =7) Mice were fasted for four hours prior to tissue harvest. Body composition was measured on anesthetized mice by dual-energy X-ray absorptiometry using a mouse densitometer (PIXImus2, Lunar Corp., Madison, WI, USA). The electrophysiological studies were conducted at Tulane University. The procedures were approved by the Institutional Animal Care and Use Committee.

### 4.2. Glucose and Insulin Tolerance Tests

Glucose and insulin tolerance tests were performed by bolus intraperitoneal injection of glucose (1 g/kg) or insulin (Humulin; Eli Lilly, IN, USA) (0.75 units/kg), respectively. Blood glucose was measured from the tail using a glucometer (OneTouch Ultra, Lifescan, PA, USA) at baseline (0) and 10, 20, 30, 45, 60, and 90 min after injection.

### 4.3. Hyperinsulinemic-Euglycemic Clamps

Before the clamp experiment, mice were fasted overnight. On the day of the clamp experiment, mice were anesthetized, and an indwelling catheter was inserted in the right internal jugular vein [49]. A three-way connector was attached to the catheter to deliver solutions intravenously. A 2 h hyperinsulinemic-euglycemic clamp was conducted in all four groups of mice with a primed (150 mU/kg body wt) and continuous infusion of insulin at a rate of 2.5, 5 or 10 mU/kg/min to raise plasma insulin within a physiological range. In total, 20% glucose was infused at variable rates to maintain glucose at basal concentrations. Blood samples (10 µL) were collected at 10 min intervals for measurement of plasma glucose concentration only. Basal and insulin-stimulated whole-body glucose turnover was estimated with a continuous infusion of [3-^3^H]glucose (PerkinElmer, Boston, MA, USA) for 2 h before the clamps (0.05 µCi/min) and throughout the clamps (0.1 µCi/min), respectively. To estimate insulin-stimulated glucose uptake in individual organs, 2-deoxy-d-[1-^14^C]glucose(2-[^14^C]-DG) was administered as a bolus (10 µCi) when euglycemic clamp had approached. Then, 10 min after 2-[^14^C]-DG being injected, tissues were taken for biochemical analysis.

### 4.4. Analysis of Liver Lipids

Neutral lipid was analyzed as described previously [50,51,52]. Frozen liver tissue was homogenized in (*v*/*v*) Folch reagent (2:1 CHCl_3_/MeOH) containing 300 μg of tritridecanoin reference standard (Nu-Check Prep Inc., Elysian, MN, USA) by bead homogenization for two cycles of 2 min at 30 Hz. Homogenates were diluted further with Folch reagent to 4 mL, treated with 800 μL of 0.9% sodium chloride solution, vortexed, and centrifuged at 4000 rpm for 5 min. The organic phase was removed and dried under N_2_ gas. Total lipids were resuspended in 330 μL of 100% chloroform and applied to HyperSep SI SPE columns (Thermo Scientific, Waltham, MA, USA) pre-equilibrated with 15 column volumes chloroform. Neutral lipids were eluted with a total of 3 mL of chloroform, dried under N_2_, and resuspended in 1 mL of methanol containing 2.5% H_2_SO_4_. Fatty acid methyl ester (FAME) production was initiated by heating at 80 °C for 1.5 h. Then, 1 mL of HPLC-grade water was added to quench the reactions, and FAMEs/cholesterol was extracted with 200 μL of hexane. A Trace 1310 gas chromotograph with a TG-5MS column (Thermo Scientific, Waltham, MA, USA) was used to separate lipids chromatographically. Lipids were detected with an ISQ single quadrupole mass spectrometer (Thermo Scientific, Waltham, MA, USA) in electron ionization mode. Masses were scanned between 40 amu and 550 amu with a scan time of 0.25 s, and temperatures were ramped from 50 °C to 290 °C in 3 stages. Xcalibur software (Thermo Scientific, Waltham, MA, USA) was used to calculate peak areas. Areas were normalized to the tritridecanoin reference standard and then to tissue weight. Histological analysis and scoring of hepatic lipids and steatosis were performed as previously described [53].

### 4.5. Lipoprotein Profile

Plasma samples (200 µL) were chromatographed via fast protein liquid chromatography (FPLC) using two Superose 6 columns in series as previously reported [54]. During size exclusion, the absorbance of the eluted samples was measured at 280 nm, allowing identification of each lipoprotein class via protein content. Fractions containing either VLDL, LDL, or HDL were pooled, and cholesterol was measured using a commercially available kit (Cayman Chemical Company, Ann Arbor, MI, USA) following procedures outlined in the package insert.

### 4.6. Quantitative Real-Time PCR

Total RNA was reverse transcribed with the iScript cDNA synthesis kit (Bio-Rad, Hercules, CA, USA). Quantitative PCR was performed using primer sets for genes of interest and reference genes (designed using NCBI’s Primer3/BLAST) and iTaq Universal SYBR Green Supermix (Bio-Rad) following the manufacturer’s protocols. Reactions were run in duplicate on an iQ5 Real-Time PCR detection system (Bio-Rad) along with a no-template control per gene. Validation experiments were performed to demonstrate that efficiencies of target and reference genes were approximately equal. Data were normalized to two reference genes (GAPDH and ACTB) using the comparative Ct method.

### 4.7. Identification of Liver-Related Neurons with PRV-152

Retrogradely transported pseudorabies viral vector (PRV-152, provided by the Center for Neuroanatomy with Neurotropic Viruses) expressing enhanced green fluorescent protein (EGFP) was used to identify liver-related neurons [17,55,56]. Under anesthesia, the liver was exposed with a small transverse incision, and ~4 μL of PRV-152 was injected into the median lobe of the liver (2 injections of 2 sites). A drop of adhesive “liquid bandage” was used to seal each injection to prevent the leakage of the virus. The animals were maintained in a biosafety level 2 facility up to 110 h post-injection.

### 4.8. Brain Slice Preparation

Acute brain slices were prepared from WT and LPL KD mice. After anesthesia with isoflurane, the brain was removed and immersed in ice-cold oxygenated artificial cerebrospinal fluid (aCSF) containing the following (in mM): 124 NaCl, 26 NaHCO_3_, 1.4 NaH_2_PO_4_, 11 glucose, 3 KCl, 1.3 MgCl_2_, 1.5 CaCl_2_, pH 7.3–7.4. Transverse hypothalamic slices containing the PVN (300 μM) were made using a vibrating microtome. The slices were stored in a holding chamber at 34–36 °C and then transferred to a recording chamber mounted on a fixed stage under an upright microscope (Eclipase, FN1, Nikon Instruments, NY, USA).

### 4.9. Whole-Cell Patch-Clamp Recordings

Whole-cell patch-clamp recordings were performed at 34–36 °C from liver-related neurons in the PVN identified under 40× water-immersion objective (N.A = 0.8). Epifluorescence was used to identify EGFP-containing neurons and infrared illumination and differential interference contrast optics (IR-DIC) to target specific cells. For whole-cell patch-clamp recordings, electrodes (3–7 M) were filled with a solution containing the following (in mM): 130 Cs+ gluconate, 10 HEPES, 5 EGTA, 1 NaCl, 1 MgCl_2_, 1 CaCl_2_, 3 CsOH, 2–3 Mg-ATP, 0.2% biocytin, pH 7.3–7.4. Electrophysiological signals were recorded using an Axoclamp 700B amplifier (Molecular Devices, San Jose, CA, USA) and acquired by pClamp (Molecular Devices, San Jose, CA, USA). Inhibitory post-synaptic currents (IPSCs) were recorded at −10 mV and excitatory post-synaptic currents (EPSCs) at −60 mV. Data were analyzed offline using pClamp or MiniAnalysis (Synaptosoft, Molecular Devices, San Jose, CA, USA).

### 4.10. Stereotaxic AVV Injection

Adeno-associated virus 8 coding for Cre recombinase and GFP under the control of the CaMKIIa promoter (AAV8-CaMKIIa-GFP-Cre, UNC viral core) was utilized to achieve recombination between flox sites of PVN neurons. Adeno-associated virus 8 coding for GFP under the control of the CaMKIIa promoter (AAV8-CaMKII-GFP, UNC viral core) was used as a control vector (mice referred to as PVN nLPL+). The 10 week old male LPL flox/flox mice were anesthetized with 2.0% isoflurane, and the surgical anesthesia plane was maintained with 1.0% isoflurane. The incision site was locally anesthetized with 100–200 μL of lidocaine (1 mg/mL). Mice were then mounted onto the stereotaxic apparatus (Neurostar, Tubingen, Germany). A total of 100 nL of the virus was injected bilaterally into the PVN (anteroposterior −0.7 mm, lateral ±0.25 mm, and dorsoventral −4.8 mm) using 0.5 μL Hamilton syringe (part# 86250, Hamilton Company, NV, USA) at a rate of 20 nL/min [57]. The syringe was kept in place for 5 min after each infusion, and the needle was withdrawn over 3 min. Two weeks following the initial injection, anesthetized mice were transcardially perfused with HBSS (with calcium and magnesium), and whole brains were fresh-frozen in liquid nitrogen-cooled 2-methylbutane, since fixed brains are incompatible with fluorescence lifetime imaging microscopy.

### 4.11. Fluorescence Lifetime Imaging Microscopy (FLIM)

FLIM was performed to detect local metabolic changes in 5–7 different areas of PVN in fresh brain sections using a Zeiss 780 laser-scanning confocal/multiphoton-excitation fluorescence microscope with a 34-Channel GaAsP QUASAR Detection Unit and non-descanned detectors for 2 photon fluorescence (Zeiss, Thornwood, NY, USA) equipped with an ISS A320 FastFLIM box and a titanium:sapphire Chameleon Ultra II (Coherent, Santa Clara, CA). The 2 photon excitation was blocked by a 2 photon emission filter. For the acquisition of FLIM images, fluorescence for nicotinamide adenine dinucleotide (NADH) and flavin adenine dinucleotide (FAD) was detected simultaneously by two photon-counting PMT detectors (H7422p-40; Hamamatsu, Japan). Images of the different areas of PVN in the brains were obtained with VistaVision software (ISS, Champaign, IL, USA) in 256 × 256 format with a pixel dwell time of 6.3 µs/pixel and averaging over 30 frames. Calibration of the system was performed by measuring the known lifetime of the fluorophore fluorescein with a single exponential decay of 4.0 ns [58]. The phasor transformation and the data analysis were carried out using Global SimFCS software (Laboratory for Fluorescence Dynamics (LFD), University of California, Irvine) as described previously [59]. The number of pixels covered with lifetimes for free and bound reduced forms of NADH and FAD were calculated in SimFCS (LFD), and the values were normalized to the total number of pixels detected as previously described [60,61].

The glycolytic index was calculated for all experimental groups using the following equation:

(1) $$\mathrm{Glycolytic}\;\mathrm{Index}\;=\;\frac{\mathrm{free}\;\mathrm{NADH}\;\mathrm{fraction}}{\mathrm{bound}\;\mathrm{to}\;\mathrm{enzyme}\;\mathrm{NADH}\;\mathrm{fraction}}$$

as defined previously [42,61].

### 4.12. Cell Culture Conditions and Reagents

mHypoE41 (N41) immortalized mouse hypothalamic neurons were purchased from CELLutions Biosystems (Winnipeg, MB, USA). N41 cells were grown in DMEM containing 1000 mg/L glucose and 10% FBS at 37 °C in the presence of 5% CO_2_. To produce cells for stable overexpression of LPL (N41 OE) or empty vector (control), N41 cells were transduced with MSCV as previously described [10]. To produce stable knockdown cells, N41 cells were transduced with shRNA (N41 553 (LPL KD)) or scrambled RNA (N41 202 (control)) containing lentivirus, as previously described [10].

### 4.13. Metabolomics

Hypothalamic cell lines described above were grown to 85% confluence. Each cell line was analyzed in triplicate. Frozen cell pellets were extracted at 2 × 10^6^ cells/mL in ice-cold lysis/extraction buffer (methanol:acetonitrile:water 5:3:2 *v*/*v*/*v*). Ultra HPLC (UHPLC)-MS-based high throughput metabolomics was performed at the University of Colorado School of Medicine Metabolomics Core. Metabolites were separated using a 9 min C18-based gradient method as previously described [62], using a Thermo Vanquish UHPLC coupled to a Thermo Q Exactive mass spectrometer. In brief, extracts (10 μL) were resolved in a Kinetex C18 column using a 3 min isocratic gradient at 250 μL/min (mobile phase: 5% acetonitrile, 95% 18 MOhm H_2_O, 0.1% formic acid) or a 9 min gradient (5% B for the first 2 min, 5%–95% B over 1 min, hold at 95% for 2 min, 95%–5% B over 1 min, re-equilibrate for 3 min). Quality control was performed via the assessment of a technical mix injected after every 10 samples as well as by comparison of internal standards. Metabolite assignments were performed with the software MAVEN [63] upon conversion of .raw files into the mzXML format using MassMatrix). Assignments were further confirmed by chemical formula determination from isotopic patterns, accurate intact mass, and retention time comparison against an in-house standard library (Sigma-Aldrich, IROA Technologies, Sea Girt, NJ, USA). Pathway analysis and analysis of metabolite set enrichment was performed using MetaboAnalyst using the positively regulated metabolites for either the LPL OE or the LPL KD lines, normalized to the respective controls [64,65].

### 4.14. Statistical Analysis

For electrophysiological experiments, continuous recordings have been conducted, and 2 min periods were analyzed with MiniAnalysis (Synaptosoft) to measure peak amplitude and frequency of post-synaptic currents. Comparison between groups was made with an unpaired two-tailed Student’s *t*-test. For all analyses, *p* < 0.05 was considered significant. Numbers are reported as the mean with standard error of the mean (SEM) unless otherwise described.

Two-way repeated measure ANOVA was performed for all age and time-related analyses, using posthoc multiple comparisons with Bonferroni correction and using Prism 8 data analysis and graphing software (GraphPad, San Diego, CA, USA), with *p* < 0.05 being considered significant and *p* < 0.1 a trend.

For metabolomics data, the values for each metabolite for either the LPL OE or the LPL KD N41 cells were normalized for their respective control line (empty vector or scrambled RNA). Differences between the mean of LPL OE vs. LPL KD were analyzed by Student’s *t*-test with *p* < 0.05 being considered significant.

## 5. Conclusions

The path to T2DM involves increased hepatic lipid accumulation, hepatic insulin resistance, and increased EGP. Since the ANS can regulate hepatic metabolism, it offers a novel therapeutic target for NAFLD and T2DM. However, mechanisms related to neuronal inputs to the ANS control of hepatic metabolism remain unclear. Here, we showed that mice with neuronal LPL deficiency and obesity showed improvements in glucose tolerance with aging. Moreover, these changes were associated with reduced hepatic lipid content and altered synaptic activity of liver-related neurons in the PVN of the hypothalamus. Loss of LPL had a marked effect on neuronal metabolism and could shift substrate utilization towards glucose oxidation in the absence of LC-PUFA transport. Our findings suggest that neuronal LPL is a component of liver-related preautonomic neurons and highlight the modulation of neuronal lipid metabolism as an intervention strategy to preserve insulin sensitivity in the aged and the obese.

## Supplementary Materials

The following are available online at https://www.mdpi.com/2218-1989/10/10/385/s1, Figure S1: Analysis of metabolites in the glycolysis pathway in immortalized hypothalamic neurons either over expressing lipoprotein lipase (LPL) (LPL OE) versus empty vector control cells (Emp) and LPL knock-down cells (LPL KD) versus 202 control cells (202 control). Figure S2: Analysis of metabolites in the TCA pathway in immortalized hypothalamic neurons either over expressing lipoprotein lipase (LPL) (LPL OE) versus empty vector control cells (Emp) and LPL knock-down cells (LPL KD) versus 202 control cells (202 control). Figure S3: Analysis of fatty acid metabolites in immortalized hypothalamic neurons either over expressing lipoprotein lipase (LPL) (LPL OE) versus empty vector control cells (Emp) and LPL knock-down cells (LPL KD) versus 202 control cells (202 control). Figure S4: Pathway enrichment analysis (MetaboAnalyst) of positively regulated metabolites in immortalized hypothalamic neurons either over expressing lipoprotein lipase (LPL) (LPL OE) or with depleted LPL (LPL KD). Figure S5: Metabolite set enrichment analysis (MetaboAnalyst) of positively regulated metabolites in immortalized hypothalamic neurons overexpressing either lipoprotein lipase (LPL) (LPL OE) or in LPL knock-down cells (LPL KD).
