# Supplementary material for: Neuronal Lipoprotein Lipase Deficiency Alters Neuronal Function and Hepatic Metabolism
Source: Metabolites. 2020 Sep 28;10(10):385. doi: 10.3390/metabo10100385 (PMC7600143; doi:10.3390/metabo10100385)
Supplement: Supplementary file 1 [file metabolites-10-00385-s001.pdf]

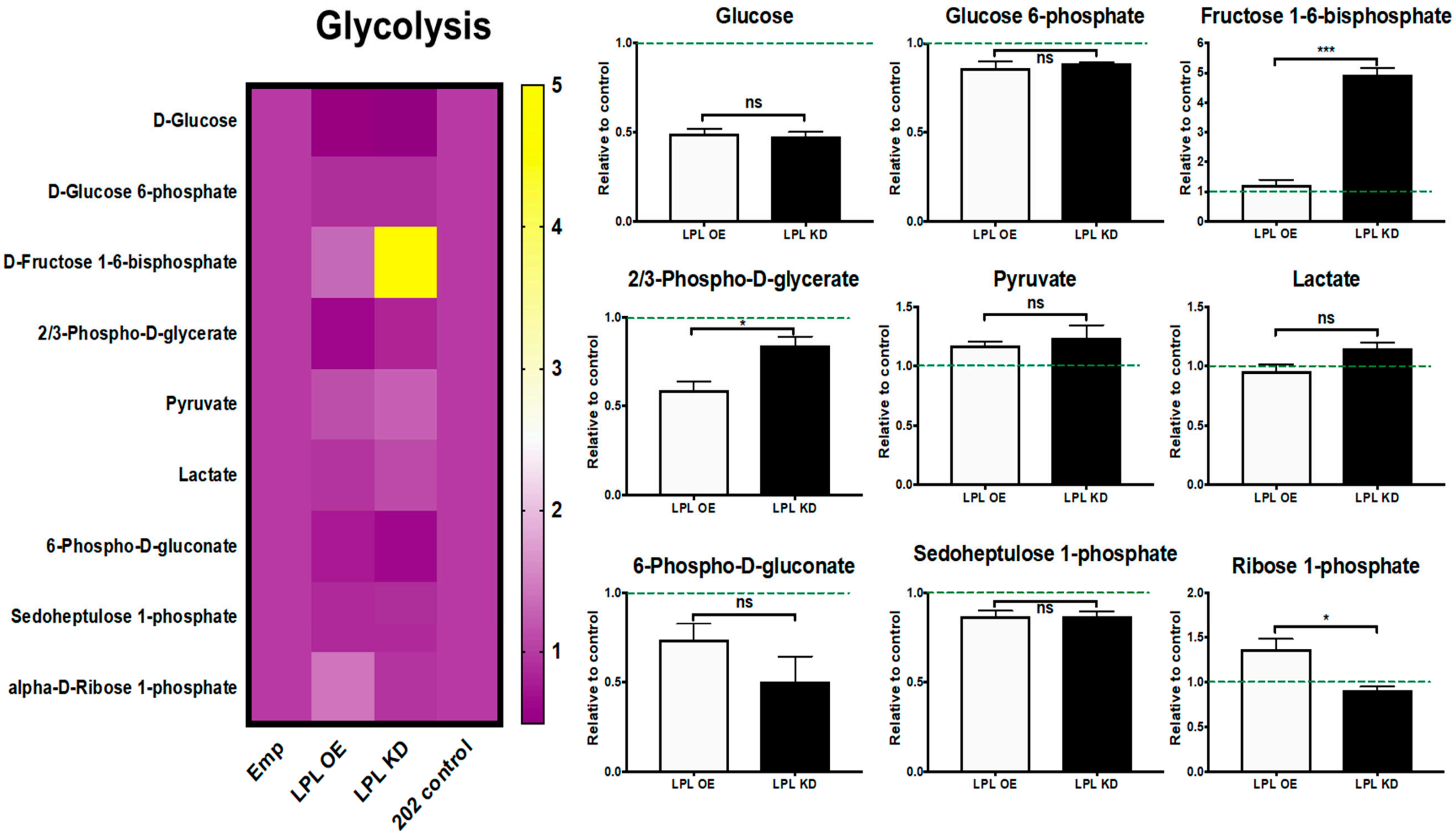

Figure S1. Analysis of metabolites in the glycolysis pathway in immortalized hypothalamic neurons either over expressing lipoprotein Lipase (LPL) (LPL OE) versus Empty vector control cells (Emp), or in LPL knock-down cells (LPL KD) versus 202 control cells (202 Control).

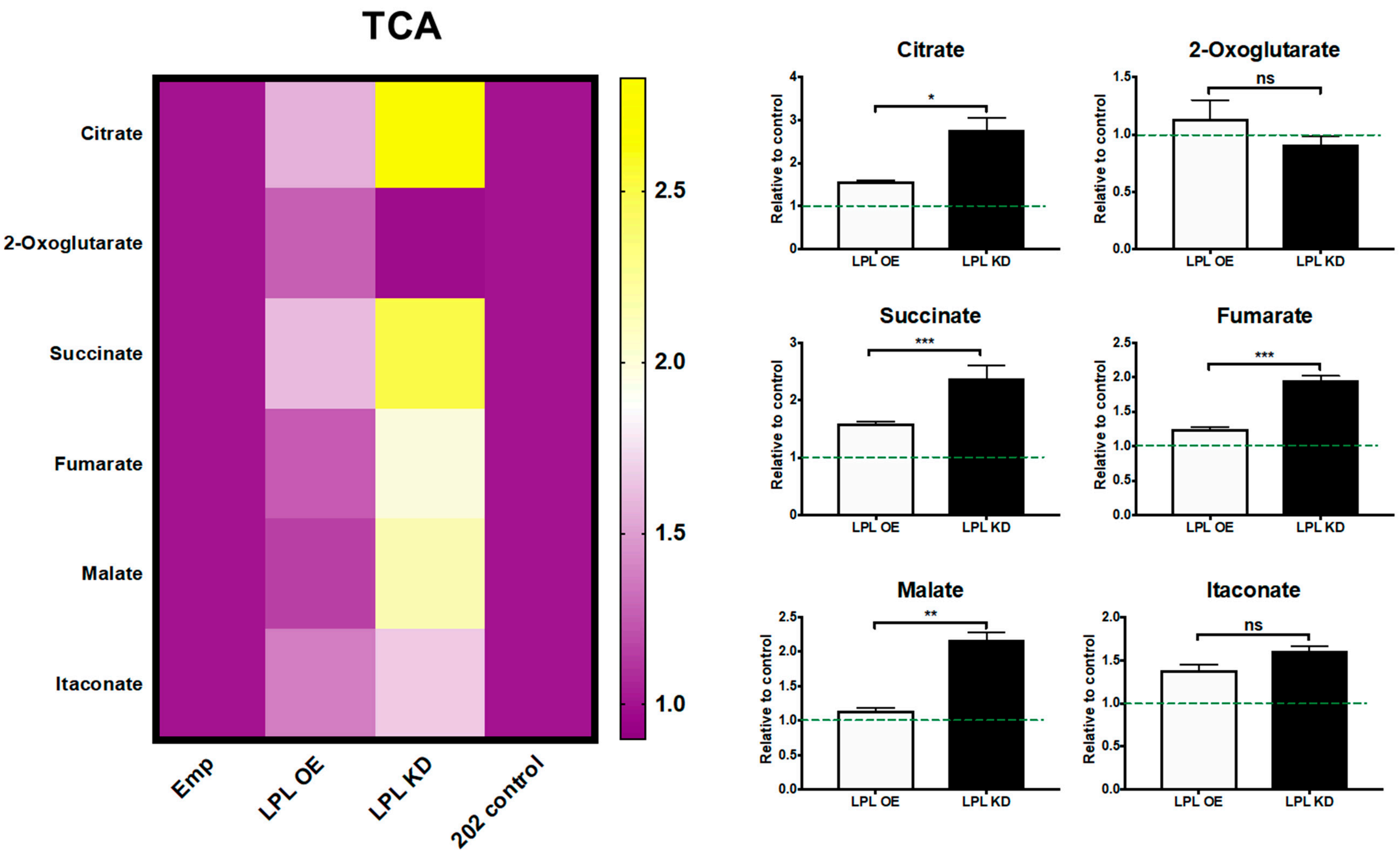

Figure S2. Analysis of metabolites in the TCA pathway in immortalized hypothalamic neurons either over expressing lipoprotein Lipase (LPL) (LPL OE) versus Empty vector control cells (Emp), or in LPL knock-down cells (LPL KD) versus 202 control cells (202 Control).

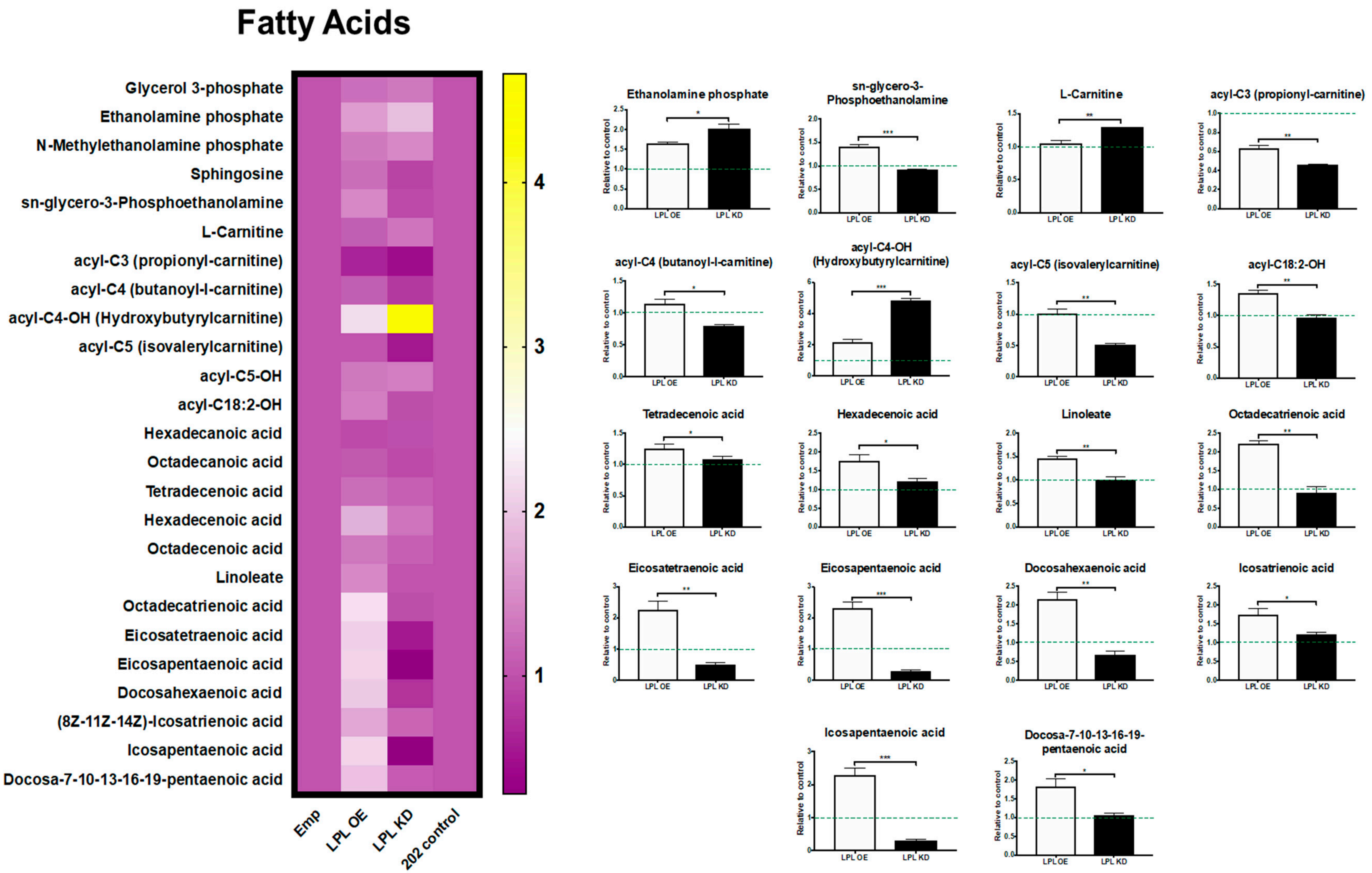

Figure S3. Analysis of fatty acid metabolites in immortalized hypothalamic neurons either over expressing lipoprotein Lipase (LPL) (LPL OE) versus Empty vector control cells (Emp), or in LPL knock-down cells (LPL KD) versus 202 control cells (202 Control).

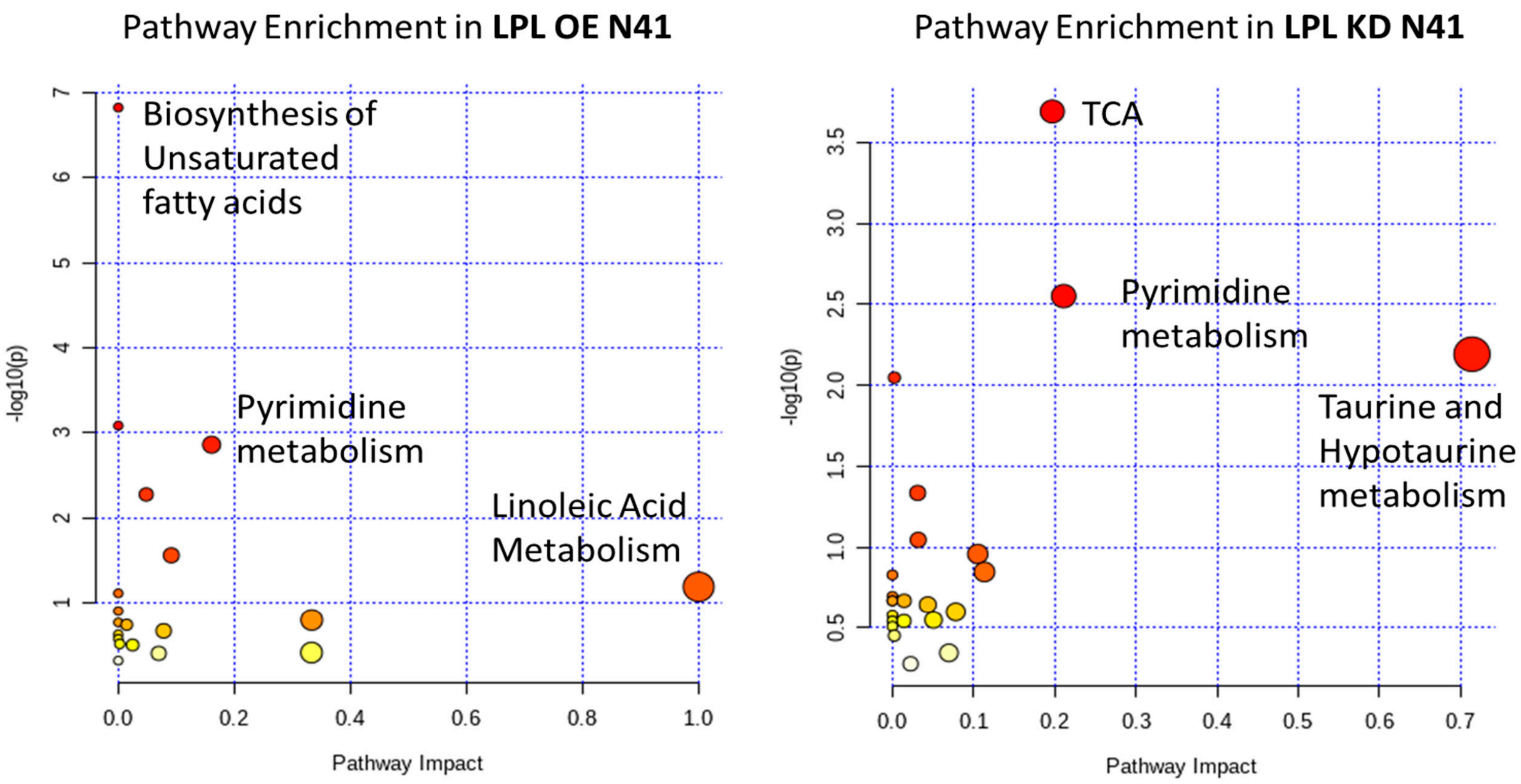

Figure S4. Pathway enrichment analysis (MetaboAnalyst) of positively regulated metabolites in immortalized hypothalamic neurons either over expressing lipoprotein Lipase (LPL) (LPL OE), or with depleted LPL (LPL KD).

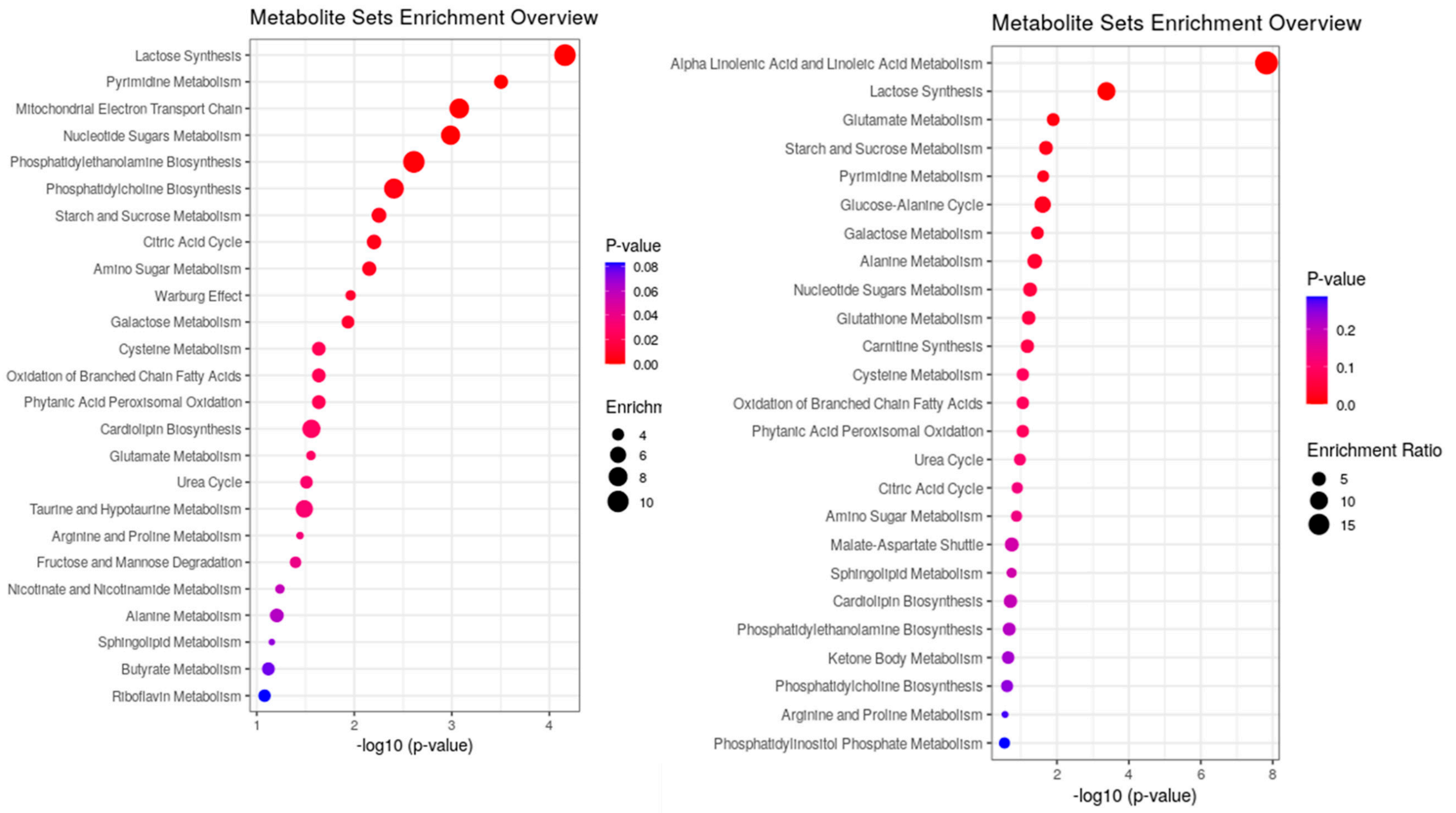

Figure 5. Metabolite Set Enrichment analysis (MetaboAnalyst) of positively regulated metabolites in immortalized hypothalamic neurons either over expressing lipoprotein Lipase (LPL) (LPL OE), or in LPL knock-down cells (LPL KD).

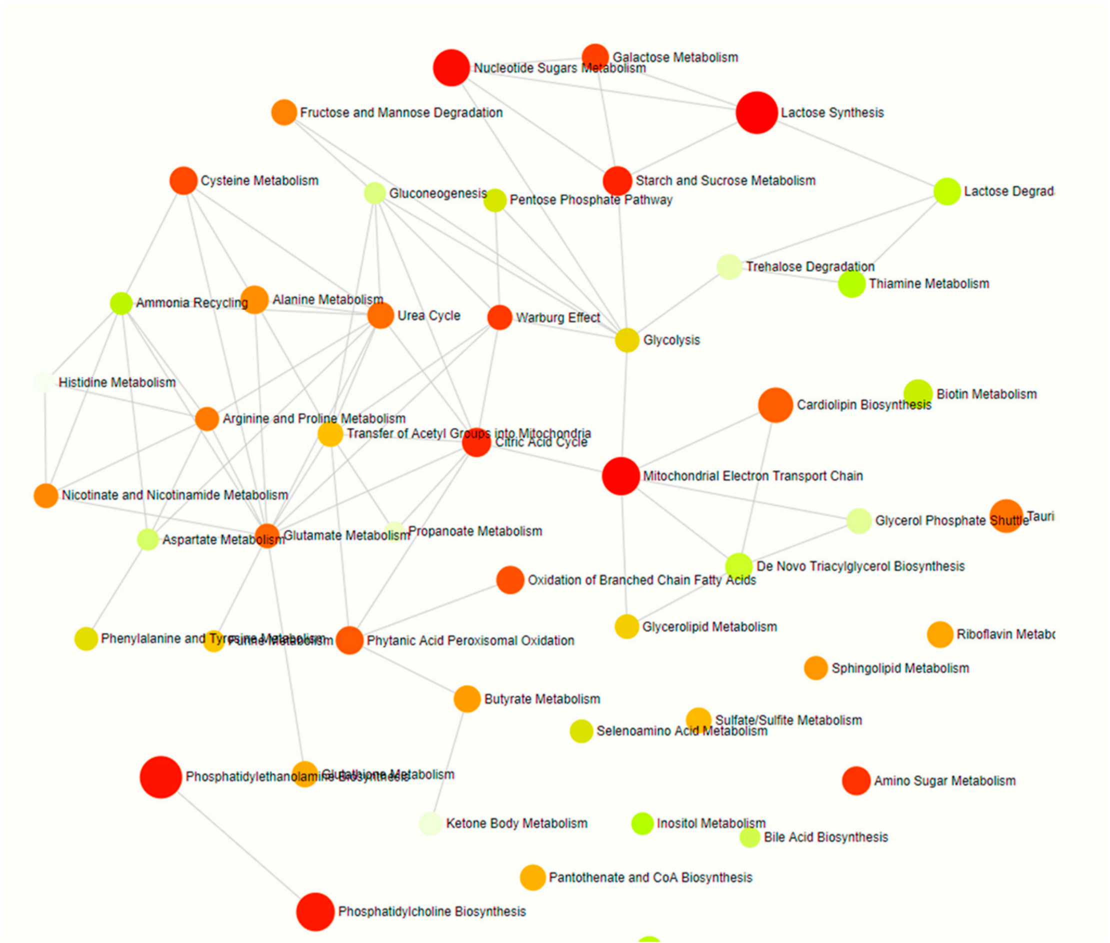

Figure S6. Metabolite Set Enrichment map (MetaboAnalyst) of positively regulated metabolites in immortalized hypothalamic LPL knock-down cells (LPL KD).
